# Data on the role of starch and ammonia in green synthesis of silver and iron oxide nanoparticles

**DOI:** 10.1016/j.dib.2016.03.068

**Published:** 2016-03-26

**Authors:** Seyedeh Masumeh Ghaseminezhad, Seyed Abbas Shojaosadati

**Affiliations:** a Nanomaterials Group, Engineering faculty, Tarbiat Modares University, P.O. Box 14155-114, Tehran, Iran; b Biotechnology Group, Chemical Engineering Faculty, Tarbiat Modares University, Iran

**Keywords:** Starch, Iron oxide, Silver nanoparticles, Alkaline condition

## Abstract

Green synthesis of nanoparticles by using starch has recently attracted considerable attention due to their biodegradability, non-toxicity, and cost effectiveness. The data presented in this article are related to the article entitled “Evaluation of antibacterial activity of Ag/Fe_3_O_4_ nanocomposites synthesized by using starch” (Ghaseminezhad and Shojaosadati, 2016) [1]. Here, Fe_3_O_4_ nanoparticles and silver nanoparticles were synthesized by using starch under alkaline condition. Hydrodynamic diameter of starch and starch coated silver nanoparticles were determined under heat treatment and different pH. This data also display absorbance peak of silver nanoparticles synthesized by starch under different pH conditions (6.5, 8, and 10). Iodometric titration confirmed that both components of starch (amylose and amylopectin) can adsorb on the surface of Fe_3_O_4_ nanoparticles.

**Specifications Table**

**Table:** 

Subject area	Chemistry, Biology, and material science
More specific subject area	Green synthesis of Ag/Fe_3_O_4_ nanocomposite by using starch
Type of data	Figures
How data was acquired	UV–visible absorption spectra of samples were obtained by a double beam UV–visible spectrophotometer (Cary 100, Varian) at a resolution of 1 nm in the range 200–800 nm.The size distribution and average size of samples were determined by dynamic light scattering (DLS) (Malvern, UK).
Data format	Analyzed
Experimental factors	Synthesis of Ag and Fe_3_O_4_ nanoparticles by using starch under alkaline condition
Experimental features	Effect of pH on starch and silver nanoparticles synthesized by starch. Evaluation of absorption of starch component on Fe_3_O_4_ nanoparticles.
Data source location	Chemical Engineering Faculty, Tarbiat Modares University, Tehran, Iran.
Data accessibility	Data are available with this article.

**Value of the data**

• Data regarding the heat and alkaline treatment on starch structure will be useful for size-controlled synthesis of nanoparticles by starch.
• Investigation of affinity of starch components (amylose and amylopectin) to adsorb on Fe_3_O_4_ nanoparticles will be useful to exploit the mechanism of the nanoparticle formation.
• The importance of ammonia concentration in silver nanoparticles synthesized by starch. It can change pH and the number of electron to reduce silver ions.

## 1 Data

The data display the characterization of starch and silver nanoparticles synthesized by starch under different condition. Also, UV–vis spectra of Fe_3_O_4_ NPs, supernatant and starch with iodine are demonstrated.

## 2 Experimental design, materials and methods

### 2.1 Materials

Soluble starch from potato, AgNO_3_, FeCl_3_.6H_2_O, FeSO_4_.7H_2_O, NH_3_.H_2_O, potassium iodide, and iodine were purchased from Sigma Aldrich.

### 2.2 Heat and alkaline treatment of starch

**Fig. 1:**
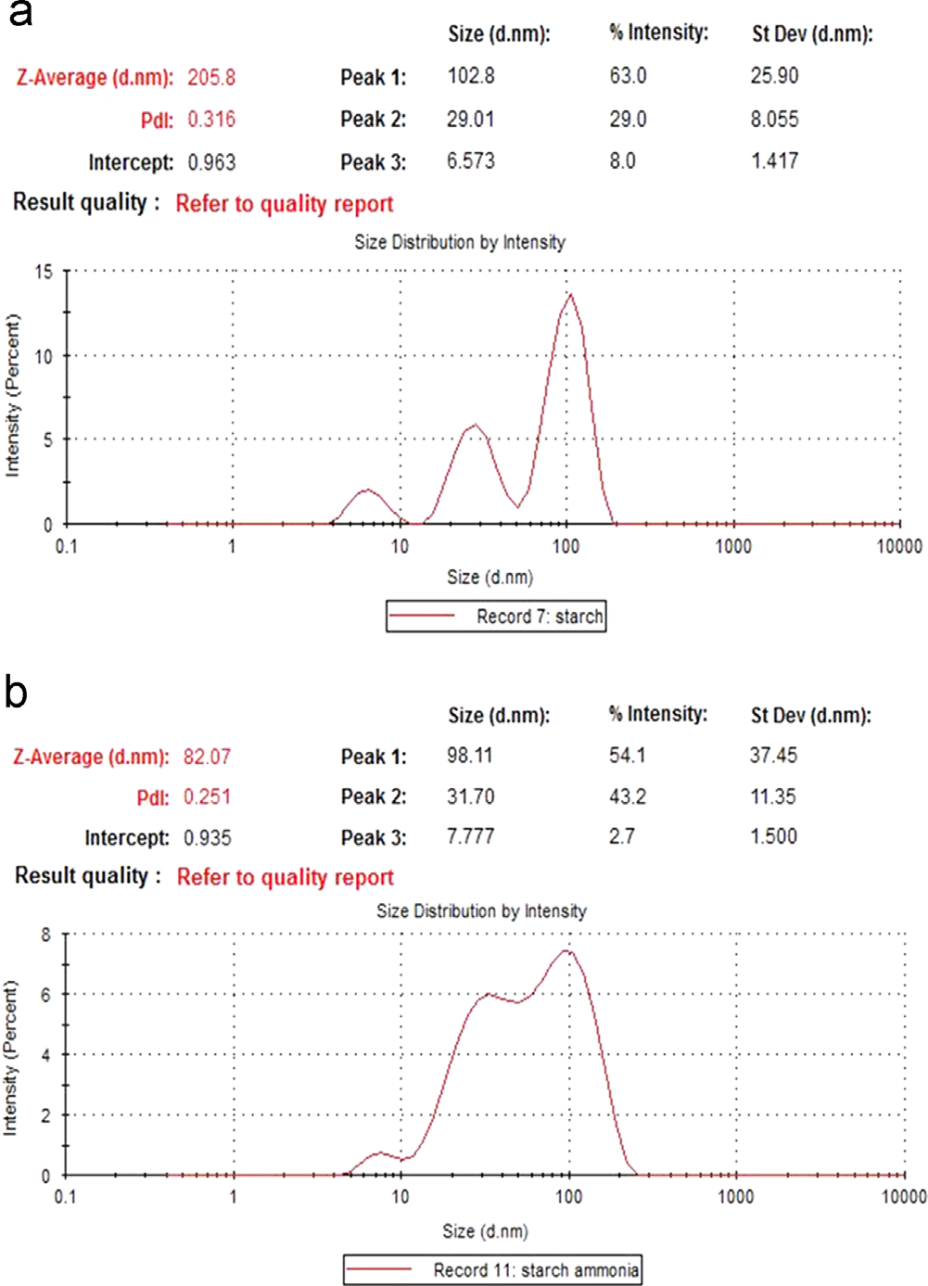
DLS analysis of starch (a) under heat treatment and (b) under heat and alkaline treatment.

Starch molecules were analyzed under heat treatment (at 95 °C for 20 min) with or without subsequent alkaline treatment by DLS. During the heating process, the starch granules first swell and then burst, the semi-crystalline structure is lost and the smaller amylose molecules start leaching out of the granules. Fig. 1(a) shows the hydrodynamic diameter of starch particles after heat treatment at 95 °C for 15 min. The poor DLS result is because of non-spherical shape of amylose and amylopectin chains and too polydispersity. As shown in Fig. 1(b), hydrodynamic diameter and size distribution of starch particles decreased by adding ammonia (pH 10) which could be related to hydrolyze amylose and amylopectin chains under alkaline medium [1], [2].

### 2.3 Evaluation of starch adsorption on Fe_3_O_4_ nanoparticles

Fe_3_O_4_ nanoparticles were synthesized according to the modification procedure previously reported [3]. Briefly FeCl_3_·6H_2_O (1.49 g) and FeSO_4_·7H_2_O (0.765 g) were added to 200 ml of the starch solution (0.6%, w/w), which was then heated at 60 °C under nitrogen atmosphere, then NH_3_·H_2_O (8 mol l^−1^) was added drop wise to reach pH 10. After 3 h, the synthesized nanoparticles were separated from the supernatant by an external magnetic field and were washed several times by deionized water. To assess which component of potato starch adsorbed on the Fe_3_O_4_ nanoparticles, the supernatant, Fe_3_O_4_ nanoparticles and starch were titrated with 0.1 N iodine solution (I_2_/KI).

**Fig. 2:**
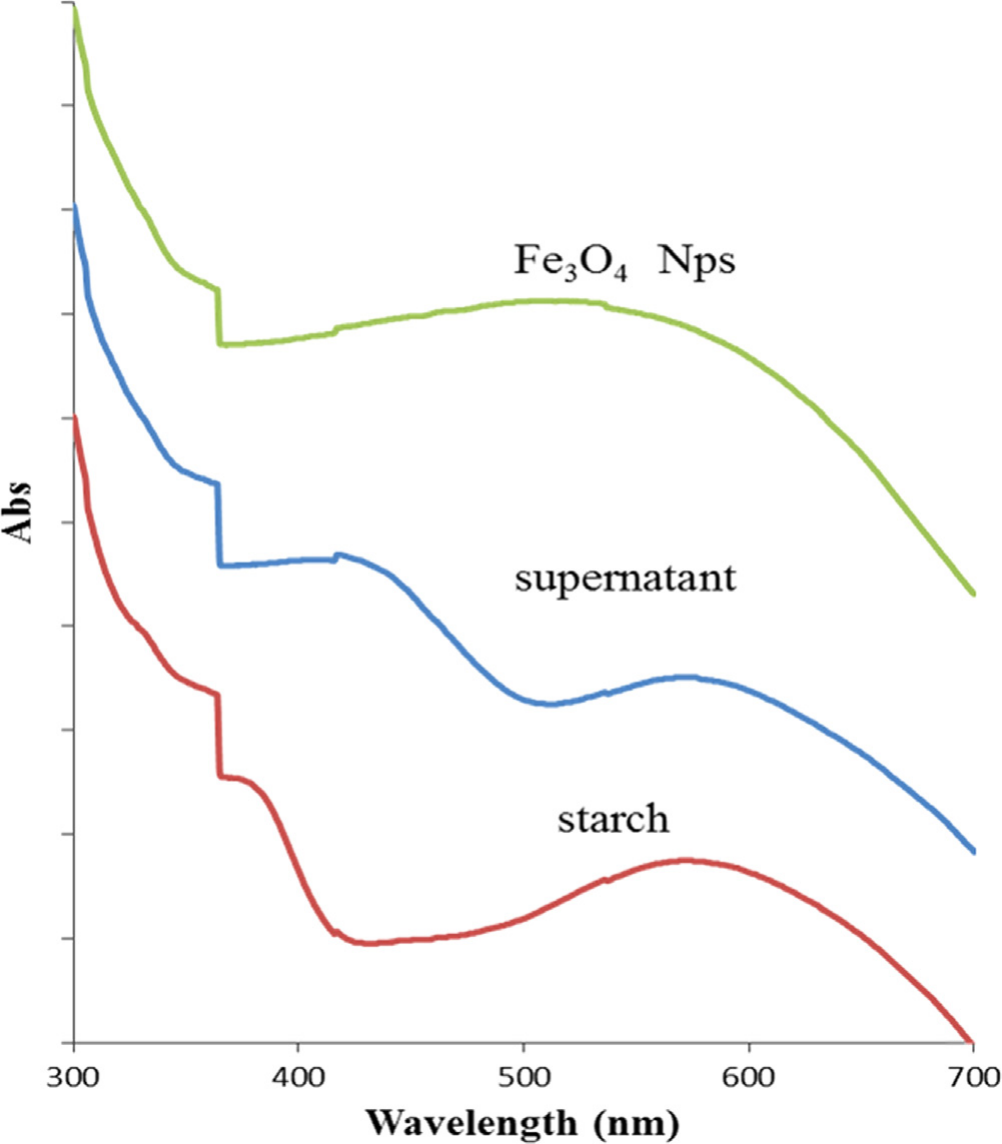
UV–vis Spectra of Fe_3_O_4_ NPs, supernatant and starch with iodine.

As shown in Fig. 2, the mixture of starch and iodine solution gave two peaks at 575 and 365 nm which are related to amylose-iodine complex and excess iodine respectively. The addition of iodine to supernatant introduced two peaks at 577 and 414 nm. These peaks can be attributed to amylose-iodine complex and iron ions-iodine complex, respectively. The mixture of Fe_3_O_4_ and iodine solution has broad peak at 530 nm. It can be attributed to the overlap between absorbance of starch (amylose and amylopectin)–iodine and iron–iodine complexes.

### 2.4 Synthesis and characterization of starch coated silver nanoparticles under different pH conditions

**Fig. 3:**
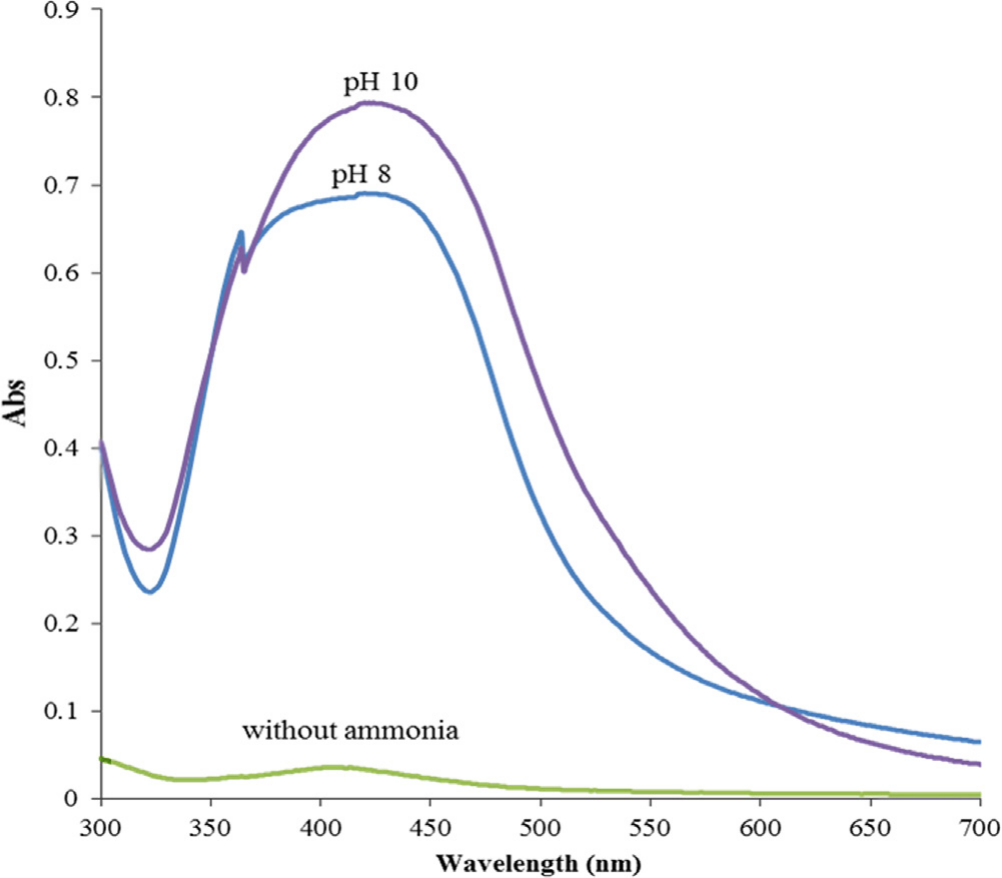
UV–vis spectra of silver nanoparticles under different pH conditions.

**Fig. 4:**
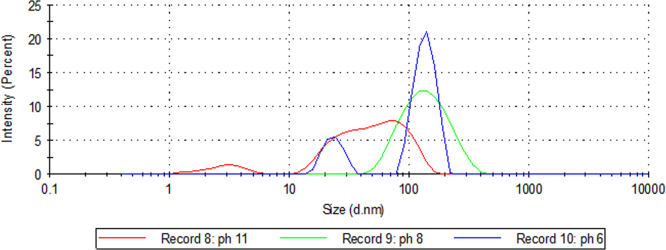
DLS analysis of starch coated silver nanoparticles under different pH conditions.

In order to synthesis of silver nanoparticles, AgNO_3_ (0.3 g) was added to 100 ml of the starch solution (1%, w/w) then the pH was adjusted (6.5, 8, 10) by ammonia, it was heated for one hour at 80 °C. As shown in Fig. 3, SPR band of silver nanoparticles is too weak in the absence of ammonia. DLS also shows that average size and size distribution of silver nanoparticles decrease by increasing pH (Fig. 4). This confirms the result obtained by UV–vis spectroscopy and our previous study [1].
